# Carboxylesterase 1d (Ces1d) does not contribute to cholesteryl ester hydrolysis in the liver

**DOI:** 10.1016/j.jlr.2021.100093

**Published:** 2021-06-18

**Authors:** Jihong Lian, Jelske N. van der Veen, Russell Watts, René L. Jacobs, Richard Lehner

**Affiliations:** 1 Group on Molecular and Cell Biology of Lipids, University of Alberta, Edmonton, Alberta, Canada; 2 Department of Pediatrics, University of Alberta, Edmonton, Alberta, Canada; 3 Department of Agricultural, Food and Nutritional Science, University of Alberta, Edmonton, Alberta, Canada; 4 Department of Cell Biology, University of Alberta, Edmonton, Alberta, Canada

**Keywords:** HDL, VLDL, lipase activity, cardiovascular disease, cholesterol metabolism, liver, reverse cholesterol transport, carboxylesterase, cholesteryl ester hydrolase, Western-type diet, AADAC, arylacetamide deacetylase, Ces1d, carboxylesterase 1d, CYP7A1, cholesterol 7α-hydroxylase, FC, free cholesterol, HSL, hormone-sensitive lipase, LD, lipid droplet, LKO, liver-specific Ces1d KO, Lox, *Ces1d*^*flox/flox*^, 4-MU, 4-methylumbelliferone, RCT, reverse cholesterol transport, SHP, small heterodimer partner, SR-BI, scavenger receptor class B type I, TC, total cholesterol, WAT, white adipose tissue, WTD, Western-type diet

## Abstract

The liver is the central organ regulating cholesterol synthesis, storage, transport, and elimination. Mouse carboxylesterase 1d (Ces1d) and its human ortholog CES1 have been described to possess lipase activity and play roles in hepatic triacylglycerol metabolism and VLDL assembly. It has been proposed that Ces1d/CES1 might also catalyze cholesteryl ester (CE) hydrolysis in the liver and thus be responsible for the hydrolysis of HDL-derived CE; this could contribute to the final step in the reverse cholesterol transport (RCT) pathway, wherein cholesterol is secreted from the liver into bile and feces, either directly or after conversion to water-soluble bile salts. However, the proposed function of Ces1d/CES1 as a CE hydrolase is controversial. In this study, we interrogated the role hepatic Ces1d plays in cholesterol homeostasis using liver-specific Ces1d-deficient mice. We rationalized that if Ces1d is a major hepatic CE hydrolase, its absence would (1) reduce *in vivo* RCT flux and (2) provoke liver CE accumulation after a high-cholesterol diet challenge. We found that liver-specific Ces1d-deficient mice did not show any difference in the flux of *in vivo* HDL-to-feces RCT nor did it cause additional liver CE accumulation after high-fat, high-cholesterol Western-type diet feeding. These findings challenge the importance of Ces1d as a major hepatic CE hydrolase.

The liver is an important organ in maintaining whole-body cholesterol homeostasis by mediating cholesterol synthesis, uptake, storage (in lipid droplets [LDs]), transport (VLDL), and elimination (bile). Accumulation of excessive cholesterol in the peripheral tissues such as the arteries drives pathological consequence including atherosclerosis (1). Reverse cholesterol transport (RCT) is a pathway by which cholesterol is removed from peripheral tissues and delivered to the liver, where it is excreted in bile, and subsequently into the feces in the form of bile acid and free cholesterol (FC). HDL acts as the acceptor of cholesterol effluxed from peripheral tissues and the transport vehicle for delivery of cholesterol to the liver. The majority of effluxed cholesterol is esterified to cholesteryl ester (CE) by LCAT (2). Scavenger receptor class B type I (SR-BI) then mediates selective uptake of CE from HDL particles into the liver. However, the detailed process downstream of CE uptake by SR-BI in hepatocytes is not fully understood.

It has been reported that in the liver, CE derived from HDL requires neutral CE hydrolase activity for conversion to FC (3). FC can then be secreted into bile (either as FC or after conversion to bile acids) or esterified by ACAT to CE, which is stored in LDs or secreted in VLDL. Mobilization of CE stored in LDs also requires participation of CE hydrolase(s). Despite the important roles of CE hydrolysis in the last step of RCT and in the liver cholesterol homeostasis, the CE hydrolase(s) that is/are involved in these biological processes in the liver still remain(s) to be determined.

The roles of carboxylesterases, including carboxylesterase 1d (Ces1d, previously annotated as Ces3 or TGH, human ortholog CES1), in lipid metabolism have been studied extensively (reviewed in (4, 5)). In the liver, Ces1d/CES1 has been shown to participate in triacylglycerol (TAG) metabolism and the mobilization of preformed TAG for VLDL assembly (6, 7, 8). Loss of Ces1d in mice enhances insulin sensitivity and protects from high-fat diet–induced liver steatosis by increasing FA oxidation and decreasing hepatic de novo lipogenesis (6, 8).

It has been proposed that CES1 catalyzes CE hydrolysis in the human macrophage (9, 10) and that Ces1d/CES1 catalyzes CE hydrolysis in the liver (11, 12, 13), thereby promoting cholesterol removal from the body by the RCT pathway. Excess cholesterol in peripheral tissues and macrophages is esterified to CE and stored in LDs. This storage of CE is initially beneficial to avoid cell toxicity from FC, but excessive accumulation of CE in macrophages leads to the formation of foam cells. Foam cells accumulated in the intima of arterial walls promote the development of atherosclerotic lesions. Hydrolysis of CE in macrophages is the rate-limiting step of cholesterol efflux, which is the first step of RCT (14). It is important to note that mouse macrophages do not express Ces1d, suggesting Ces1d does not play a critical role in mouse macrophage CE metabolism. Specific expression of CES1 in mouse macrophages was reported to reduce atherosclerosis in *Ldlr*^*−/−*^ mice (9). However, the CE hydrolase activity of Ces1d/CES1 and the role of CES1 in macrophage CE turnover have been challenged (15, 16, 17). In line with these studies, it has been also reported that CES1 knockdown in human macrophages did not reduce cholesterol efflux, but decreased cholesterol uptake by attenuating CD36 and scavenger receptor-A (SR-A) expression (18, 19), thereby preventing atherosclerosis development.

The controversial studies on the role of CES1 as a CE hydrolase in cholesterol efflux from macrophages also question the function of Ces1d/CES1 in liver cholesterol metabolism. In one study, overexpression of CES1 in the liver increased bile acid content in gallbladder bile and enhanced the output of [^3^H]cholesterol from macrophages to bile and feces in the form of bile acid (11). In line with this, ablation of *Ces1d* expression in the liver of *Ldlr*^*−/−*^ mice was reported to decrease HDL-to-feces RCT indicated by decreased flux of radioisotope derived from HDL-cholesterol to fecal cholesterol and bile acid, and increased atherosclerotic lesions in the aortic arch (13). In contrast, in another study, it was shown that ablation of *Ces1d* expression in *Ldlr*^*−/−*^ mice alleviated Western-type diet (WTD)-induced atherosclerosis (20). These contradictory observations lead us to re-examine the role of Ces1d in hepatic cholesterol homeostasis.

By utilizing liver-specific Ces1d-KO mice (LKO), we assessed the effect of Ces1d inactivation on in vivo RCT and liver cholesterol storage. The results suggest that ablation of Ces1d in the liver did not alter cholesterol metabolism and challenge the proposed role of Ces1d in hydrolysis of hepatic CE.

## Materials and Methods

### Animals

All animal procedures were conducted in compliance with protocols approved by the University of Alberta’s Animal Care and Use Committee and in accordance of the Canadian Council on Animal Care policies and regulations. *Ces1d*^*flox/flox*^ (Lox) and LKO mice were generated previously (21). Animals were maintained on a 12-h light (7 am - 7 pm)/12-h dark (7 pm–7 am) cycle, controlled for temperature and humidity, and were fed a chow diet (5% fat (w/w) and 0.04% cholesterol) (PicoLab Rodent Diet). In a separate cohort, 10-week-old male mice were fed a high-fat, high-cholesterol WTD (42% kcal from fat, 0.2% cholesterol, Envigo TD 88137) for 2 weeks. Tissues were collected after 5-h fasting.

### Cell culture and generation of McArdle RH-7777 cell lines stably expressing Ces1d

McArdle RH-7777 (McA) cells were obtained from the ATCC and cultured in DMEM containing 50 units/ml penicillin/streptomycin, 10% horse serum, and 10% FBS at 37°C in humidified air containing 5% CO_2_. Wild-type McA cells were transfected with empty vector pCI-neo or with Ces1d-cDNA construct cloned into pCI-neo using Lipofectamine 2000. Transfected cells were grown in media containing 1.6 mg/ml G-418 for 5 days to select for neomycin resistance. Individual clones were isolated and analyzed for Ces1d protein by immunoblotting. Stable cell lines, designated pCI-neo and Ces1d, were thereafter maintained in media containing 0.4 mg/ml G-418.

### Immunoblot analyses

Proteins in cell lysates were resolved by SDS-polyacrylamide gels and transferred to PVDF membranes (catalog #IPVH00010; Millipore). Antibodies used in this study include anti-Ces1d (1:1,000 dilution, catalog # sc-374160; Santa Cruz) and anti-GAPDH (1:5,000 dilution, catalog # ab8245; Abcam). Immunoreactivity was detected by enhanced chemiluminescence and visualized by G:BOX system (SynGene).

### Measurement of CE hydrolase activity in the microsomes of Ces1d-expressing McA cells

Because Ces1d is a disulfide-bonded glycoprotein localized in the lumen of the endoplasmic reticulum (ER) (22, 23, 24, 25, 26), microsomes were isolated from cells for measurements of lipase activity. pCI-neo and Ces1d McA cells harvested from three 100 mm dishes with 80% confluence in homogenate buffer (250 mM sucrose, 20 mM Tris, 1 mM EDTA, pH 7.4) were homogenized using an isobiotec cell homogenizer (H&Y enterprise) with 0.1574 inch diameter ball using 3 ml syringes to pass cells firmly through the chamber 40 times to disrupt the cells. Cell lysates were spun at 600 *g* at 4°C for 5 min to isolate supernatants, which were then centrifugated for 15 min at 10,200 *g* at 4°C to pellet heavy membranes. The supernatants were then centrifuged at 425,866 *g* at 4°C for 15 min, and the microsomal pellet was resuspended in 0.1 M potassium phosphate buffer (pH 7.0), sonicated to release lumenal contents (including Ces1d), and used for the CE hydrolase activity assay. The CE hydrolase assay was performed as described previously (27). Mixed micelles of cholesteryl-[^14^C]oleate, phosphatidylcholine, and sodium taurocholate were prepared with the molar ratio of 1:4:2 in 0.1 M potassium phosphate buffer (pH 7.0) using a sonicator (model W-385, Heat Systems) at the setting of 2.5 for 2 × 1 min with 1-min interval, followed by 4 × 30 s with 30 s interval. Micelles containing 0.1 μmol cholesteryl-[^14^C]oleate (specific activity 400,000 disintegrations per minute/μmol) were used as the substrate for the assay with 100 ug of microsomal fractions. The total reaction volume was 1 ml in 0.1 M potassium phosphate buffer (pH 7.0) containing 189 μM sodium taurocholate. After 1 h incubation at 37°C, the reaction was stopped by addition of 3.25 ml of methanol/chloroform/heptane 3.85:3.42:2.73 (v/v/v) and 50 μl of 1 M NaOH to 0.5 ml of the reaction mixture. After centrifugation at 200 *g* for 10 min, 1 ml of the upper phase was used to measure radioactivity by liquid scintillation counting to assess the release of oleic acid from cholesteryl oleate. One unit (U) of enzyme activity corresponds to the release of 1 μmol of labeled oleate from CE per minute.

Cytosolic fractions of mouse white adipose tissue (WAT) were used as the positive control for hormone-sensitive lipase (HSL) CE hydrolase activity (28). In brief, mouse WAT was homogenized in homogenization buffer containing 1 mM DTT to make 20% homogenate. Fat-free cytosolic fraction was obtained by centrifugation of the homogenate at 100,000 *g* at 4°C for 45 min and recovery of the clear infranatant under the fat cake, and 100 μg of the cytosolic protein containing HSL was assessed for CE hydrolase activity as described above.

### 4-Methylumbelliferyl heptanoate hydrolysis assay

Lipase activity in the cell lysate and microsomal fraction was measured utilizing 4-methylumbelliferyl heptanoate as the substrate as described previously (29). The enzymatic reaction was initiated by the injection of 20 μl of 1 mM 4-methylumbelliferyl heptanoate in 20 mM Tris/HCl (pH 8.0), 1 mM EDTA, and 300 μM taurodeoxycholate to fractions containing 5 μg of protein in a 96-well plate in a final volume of 200 μl. The plate was incubated at 37°C, and the release of fluorescent 4-methylumbelliferone (4-MU) was detected with a Fluoroskan Ascent FL Type 374 (Thermo LabSystems) with excitation/emission wavelengths of 355/460 nm. Fluorescence values generated with a standard solution of 4-MU (sodium salt) were used to quantify 4-MU release.

### Radiolabeling of HDL-CE

[^3^H]Cholesteryl oleate was incorporated into purified human HDL (Calbiochem) using CETP activity in human lipoprotein-deficient serum. 5 mg HDL and 0.5 mCi [^3^H]cholesteryl oleate were added to 3 ml lipoprotein-deficient serum, and the volume was brought to 5 ml with saline. The mixture was incubated overnight at 37°C with stirring. Labeled HDL was then isolated by ultracentrifugation (d = 1.215). Lipid extraction and analysis was performed with a small aliquot of labeled HDL (HDL-[^3^H]CE) to confirm incorporation of the labeled CE into HDL. Lipids were separated by TLC, and 89% of radioactivity was confirmed to be associated with CE.

### In vivo RCT assessment

Male LKO and Lox mice maintained on chow diet were used in this study. HDL-[^3^H]CE (1.5 × 10^6^ disintegrations per minute) was administrated to each mouse via intravenous injection. Blood was collected 2 min (as the initial time-point), 1 h, 10 h, 24 h, 36 h, and 48 h after injection, and radioactivity decay in the plasma was determined. Feces were collected for 48 h. At 48 h after injection, the liver and gallbladder were collected after a 12 h fast.

### Analytical procedures

Liver lipids were extracted from liver homogenates in the presence of known amounts of phosphatidyl dimethylethanolamine as an internal standard by a modified Folch method (30). HPLC was carried out to determine liver CE and FC concentrations on an Agilent 1100 instrument (Santa Clara, CA) equipped with a quaternary pump and Alltech Evaporative Light-Scattering Detector 2000, using a modified version of the method of Abreu, Solgadi, and Chaminade (31).

In the in vivo RCT assessment, lipids extracted from liver homogenates were separated by TLC. Radioactivity in CE and FC was determined by liquid scintillation counting.

HDL and apoB-containing lipoproteins in plasma were separated by phosphotungstic acid/MgCl_2_ precipitation method (32), and the radioactivity in each fraction was measured by liquid scintillation counting. Total cholesterol (TC) and FC concentrations in plasma and the HDL fraction were determined using a diagnostic kit (WAKO Diagnostics) according to manufacturer's instructions.

Biliary cholesterol and bile acid concentrations were determined using kits (Trinity Biotech) according to manufacturer's instructions.

Feces collected for 48 h were vacuum freeze-dried, weighed, and ground into powder. Fecal neutral sterol and bile acid fractions were separated as described previously (33, 34). Samples were heated at 80°C for 2 h in alkaline methanol and then extracted 3 times with petroleum-ether by mixing for 30 s followed by centrifugation. Radioactivity associated with the neutral sterol fraction (top layer) and bile acid fraction (bottom layer) was quantified. Radioactivity recovered from each fraction was expressed relative to the injected tracer dose (total radioactivity in blood of each mouse at the initial time point after injection [2-min]).

### RNA isolation and real-time qPCR analysis

Total liver RNA was isolated using TRIzol reagent (Invitrogen). First-strand cDNA was synthesized from 2 μg total RNA using Superscript III reverse transcriptase (Invitrogen) primed by oligo (dt)_12-18_ (Invitrogen) and random primers (Invitrogen). Real-time qPCR was performed with Power SYBR® Green PCR Master Mix kit (Life Technologies) using the StepOnePlus-Real time PCR System (Applied Biosystems). Data were analyzed with the StepOne software (Applied Biosystems). Standard curves were used to calculate mRNA abundance relative to that of a control gene, cyclophilin. Real-time qPCR primers are summarized in supplemental Table S1. All primers used to assess expression of liver carboxylesterases are listed in supplemental Table S2. All primers were synthesized by Integrated DNA Technologies.

### Statistics

All values are expressed as the mean ± SEM. Differences among group means were assessed by two-way ANOVA (decay curve), one-way ANOVA followed by Bonferroni post hoc test, and unpaired *t* test for two group comparisons (GraphPad PRISM 8 software). Differences were considered statistically significant at ∗*P* < 0.05, ∗∗*P* < 0.01, ∗∗∗*P* < 0.001, and ∗∗∗∗*P* < 0.0001.

## Results

### Absence of CE hydrolase activity in Ces1d-expressing McA cells

To determine the CE hydrolase activity of Ces1d, we expressed Ces1d in rat hepatoma McA cells, and the expression of the protein was verified by immunoblotting (supplemental Fig. S1A). Because Ces1d is localized in the ER lumen (22, 23, 24, 25, 26), lumenal contents were released by sonication and detergent incubation of microsomes prepared from pCI-neo and Ces1d cells to allow access of the substrate for lipase activity measurements. pCI-neo microsomal fractions did not show lipase activity, whereas microsomal fractions isolated from Ces1d cells exhibited robust lipase activity (supplemental Fig. S1B).

Next, we measured CE hydrolase activity in microsomal fractions with radiolabeled CE as the substrate. Microsomal fractions isolated from Ces1d cells showed similar CE hydrolase activity as fractions prepared form pCI-neo cells, suggesting lack of CE hydrolase activity of Ces1d (supplemental Fig. S1C).

### Ablation of Ces1d in the liver did not affect circulating HDL-CE turnover

**Fig. 1:**
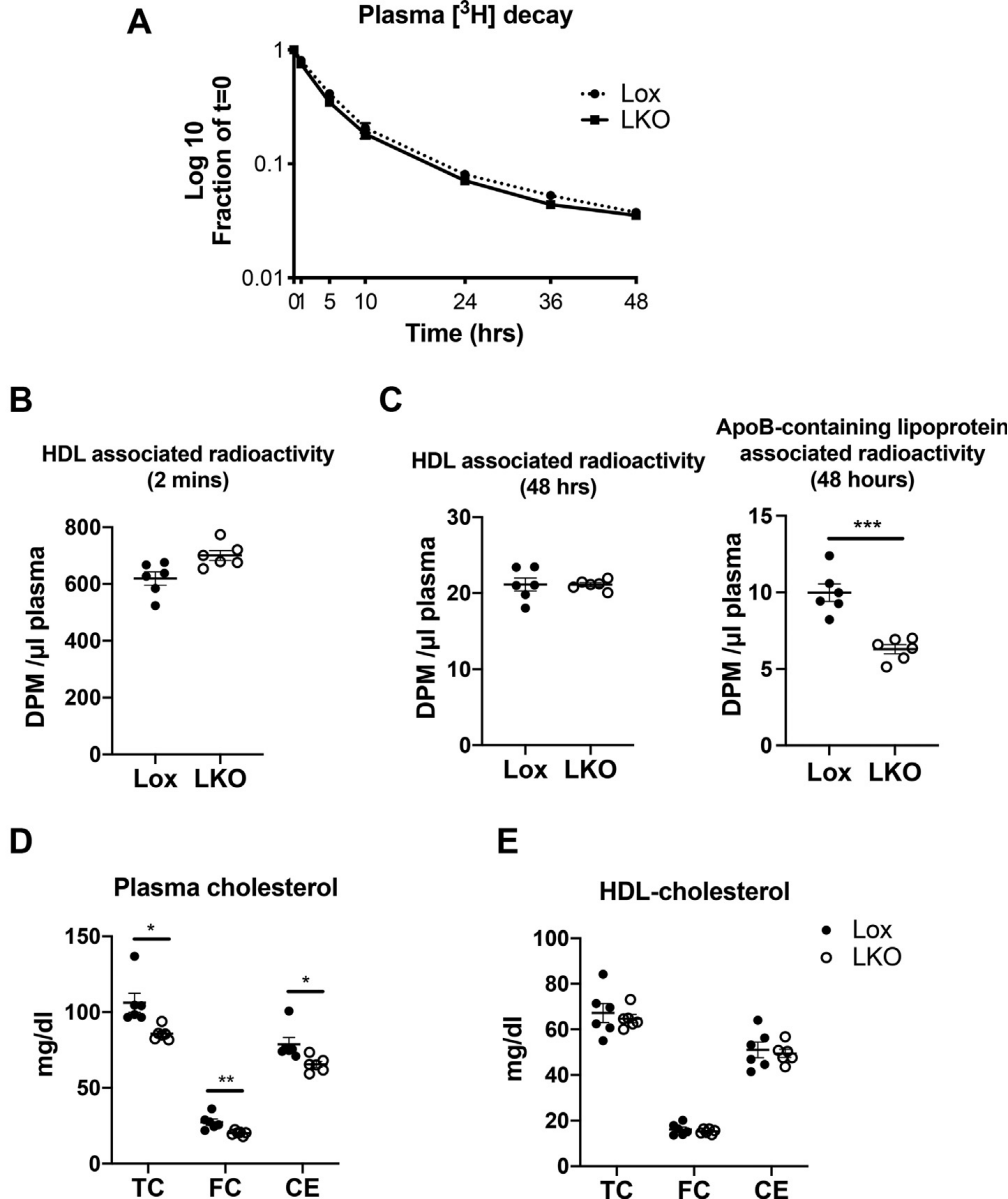
Plasma decay of HDL-CE is not affected by liver-specific ablation of Ces1d. A: Decay curve in Lox and liver-specific Ces1d-KO (LKO) mice after intravenous injection of HDL-[^3^H]CE. B and C: Radioactivity associated with HDL at the initial time point (2 min) and radioactivity associated with HDL and apoB-containing lipoprotein 48 h after HDL-[^3^H]CE injection, respectively. D: Plasma cholesterol levels. E, Plasma HDL-cholesterol levels. Lox, *Ces1d*^*flox/flox*^; Ces1d, carboxylesterase 1d.

To assess the contribution of liver Ces1d in the process of removal of HDL-derived CE from the body, in vivo RCT assay was performed by injecting HDL-[^3^H]-cholesteryl oleate into LKO mice and control Lox mice. Plasma tracer kinetics were measured over 48 h to estimate the HDL-CE turnover rate. Compared with Lox mice, ablation of Ces1d in the liver did not alter the decay rate of plasma radioactivity (Fig. 1A). Furthermore, radioactivity associated with HDL fraction in the plasma was comparable between genotypes at the initial time point and 48 h after tracer administration (Fig. 1B and C). Radioactivity associated with apoB-containing lipoproteins in plasma was decreased in the LKO mice 48 h after tracer administration (Fig. 1C), suggesting decreased cholesterol re-secretion via apoB-containing lipoproteins from HDL-derived CE. Consistent with our previous study (21), LKO mice exhibited decreased TC, FC, and CE concentrations in the plasma (Fig. 1D). Different circulating HDL-cholesterol pool size could affect specific radioactivity in HDL-CE, thus influencing the assessment of HDL-CE turnover and the uptake rate of radioactive tracer. To exclude this possibility, cholesterol levels in the plasma HDL fraction were measured, and the results showed that Lox and LKO mice had comparable HDL-TC, FC, and CE levels (Fig. 1E); thus, the decreased cholesterol level in the plasma represents decreased cholesterol associated with the fraction of apoB-containing lipoproteins. The liver is the major organ for HDL-cholesterol uptake (35); therefore, these data also suggested that the in vivo liver uptake rate of HDL-CE was not affected by Ces1d deficiency.

### Ablation of hepatic Ces1d did not alter turnover of HDL-derived CE in the liver

**Fig. 2:**
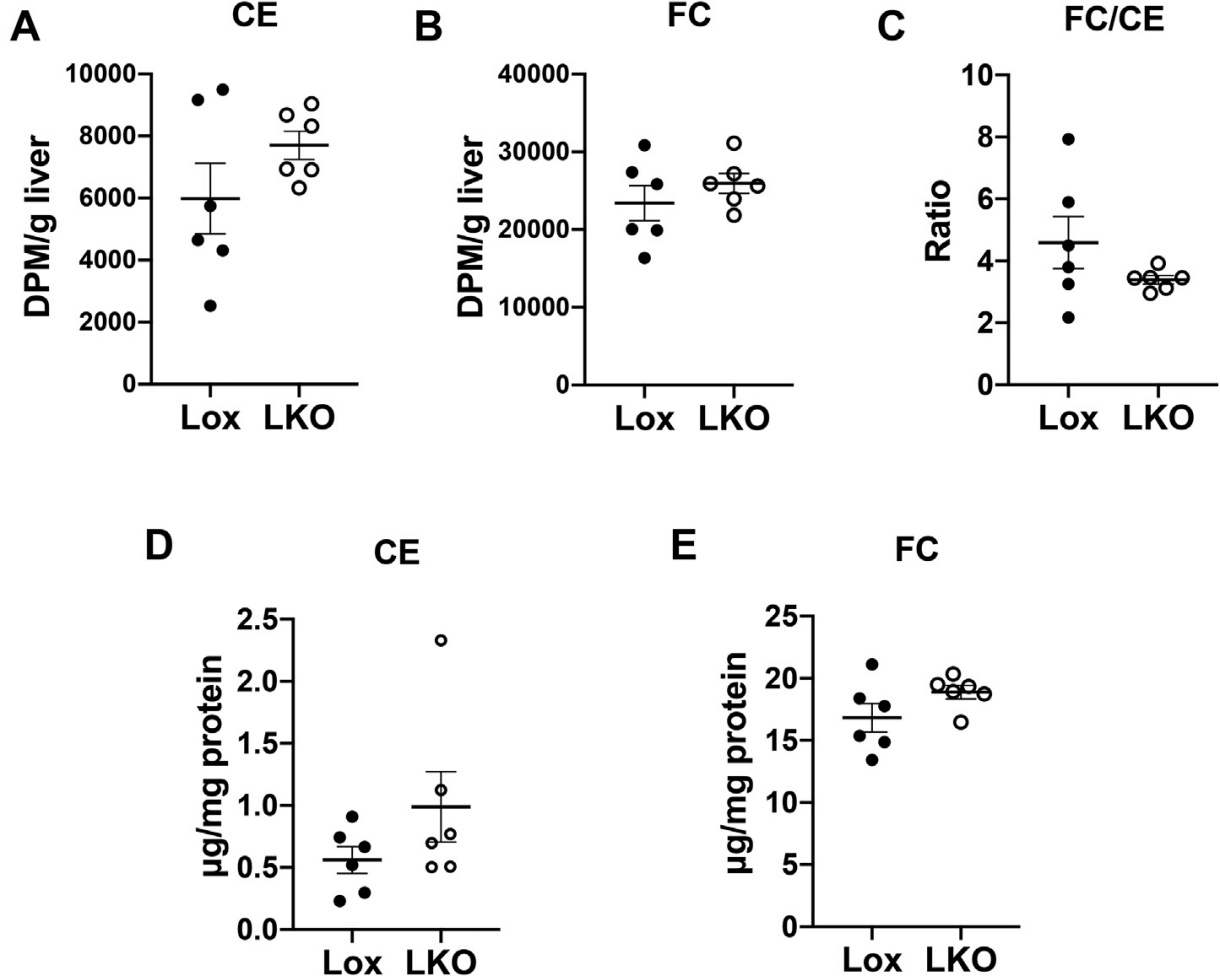
Hepatic cholesterol levels are not affected by liver-specific elimination of Ces1d. Radioactivity associated with (A) CE and (B) FC in the liver of Lox and LKO mice 48 h after HDL-[^3^H]CE injection. C: Ratio of radioactivity associated with FC to CE in the liver of Lox and LKO mice 48 h after HDL-[^3^H]CE injection. D, CE and (E) FC concentrations in the liver of Lox and LKO mice. LKO, liver-specific Ces1d KO; Lox, *Ces1d*^*flox/flox*^; Ces1d, carboxylesterase 1d; FC, free cholesterol.

To investigate whether conversion of HDL-derived CE to FC in the liver is influenced by liver Ces1d deficiency, radioactivity associated with CE and FC was measured in liver lipids. 48 h after injection of HDL-[^3^H]CE, radioactivity content in CE and FC, and FC/CE ratio, was not different between Lox and LKO mice (Fig. 2A–C), suggesting normal hepatic hydrolysis of HDL-derived CE to FC in LKO mice. Hepatic concentrations of CE and FC were not different between Lox and LKO groups (Fig. 2D, E), indicating comparable TC pool size in the liver.

### Excretion of HDL-derived cholesterol was not affected by liver Ces1d deficiency

**Fig. 3:**
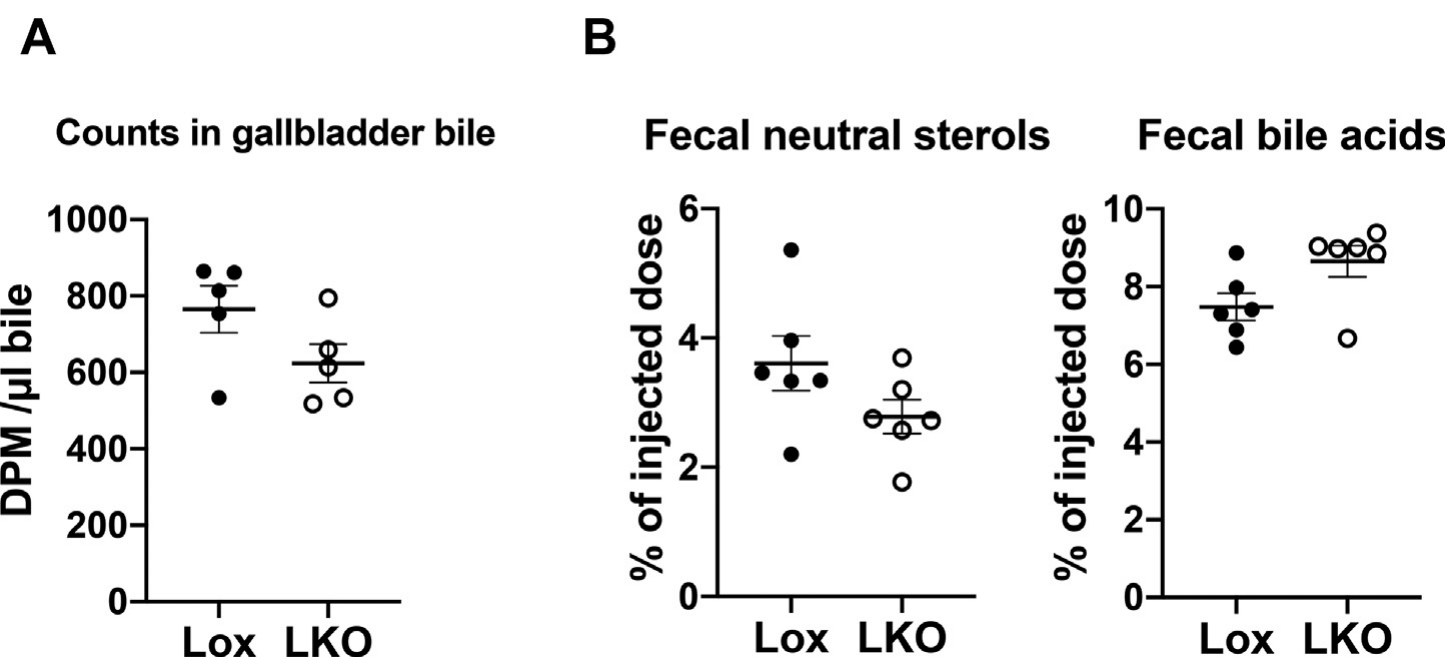
Excretion of HDL-cholesterol is not affected by liver-specific ablation of Ces1d. A: Radioactivity in the gallbladder bile. B: Excretion of fecal sterol and bile acid after 48 h of HDL-[^3^H]CE injection. Ces1d, carboxylesterase 1d.

In the final stage of RCT, HDL-cholesterol delivered to the liver is excreted into bile and subsequently the feces after conversion to bile acid or in the form of neutral sterols. Hepatic Ces1d deficiency did not alter the concentration of [^3^H] tracer in the gall bladder bile (Fig. 3A), suggesting the same amount of HDL-derived cholesterol was secreted into bile either in the form of bile acids or neutral sterols. 48 h after injection of HDL-[^3^H]CE injection, fractions of the tracer recovered in fecal neutral sterol and bile acid were analyzed (Fig. 3B). No difference was observed between the Lox and LKO groups, indicating that the overall circulating HDL-to-feces RCT cholesterol excretion was not influenced by hepatic Ces1d deficiency.

### Hepatic metabolic pathways of HDL-derived CE are intact in liver-specific Ces1d-deficient mice

**Fig. 4:**
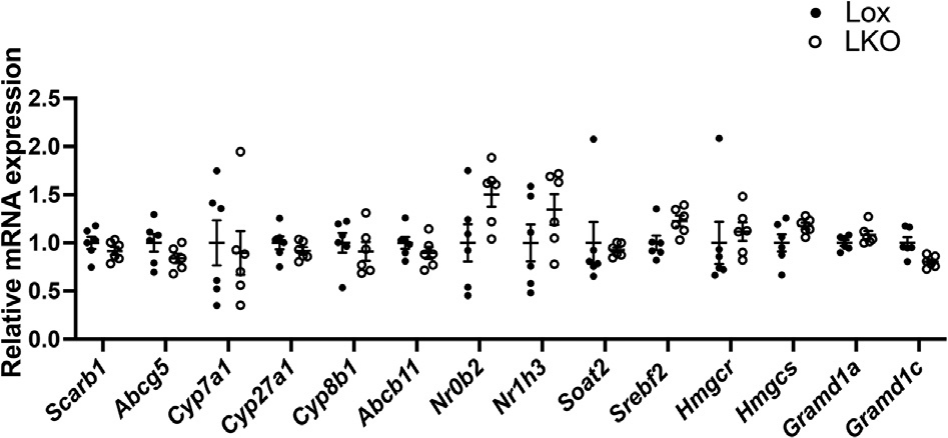
Regulation of cholesterol and bile acid metabolism in the liver of liver-specific Ces1d-KO mice.

Hepatic SR-BI mediates selective uptake of HDL-CE in the liver (36). No difference was observed in SR-BI (gene name *Scarb1*) gene expression in the livers of Lox and LKO mice (Fig. 4). The ABCG5/ABCG8 heterodimer mediates FC excretion into bile in the liver (37, 38). Increased expression of ABCG5/ABCG8 leads to enhanced biliary neutral sterol secretion and fecal neutral sterol output (39). *Abcg5* gene expression did not change in the liver of LKO mice (Fig. 4). Expression of genes encoding key enzymes in the bile acid synthesis pathway from cholesterol, cholesterol 7α-hydroxylase (*Cyp7a1*), sterol 27-hydroxylase (*Cyp27a1*), and sterol 12-alpha-hydroxylase (*Cyp8b1*) did not change in the liver of LKO mice (Fig. 4). ABCB11 (bile salt export pump) is the major transporter for bile acid secretion from hepatocytes into bile (40). No difference was observed in *Abcb11* expression between Lox and LKO mice (Fig. 4). The small heterodimer partner (SHP) is a nuclear receptor that mediates feedback inhibition of bile acid synthesis through inhibition of CYP7A1 (41). Liver X receptor (LXR) directly activates *Cyp7a1* expression, thus promoting conversion of cholesterol into bile acid (42). Expression of *Nr0b2* (encoding SHP) and *Nr1h3* (encoding LXRα) was not different between Lox and LKO mice (Fig. 4).

ACAT2 is responsible for esterification of FC to produce CE in the liver, and its gene (*Soat2*) expression was not changed in the liver of LKO mice (Fig. 4). The master transcriptional regulator of cholesterol de novo synthesis SREBP2 (encoded by *Srebf2*), and its target genes *Hmgcr* (encoding HMG-CoA reductase) and *Hmgcs* (encoding HMG-CoA synthase), did not show differences in gene expression between Lox and LKO mice (Fig. 4). Activation of SREBP2 is controlled by the ER cholesterol concentration through SREBP-SCAP-INSIG protein complex (43). The comparable gene expression in SREBP2-regulated genes suggested that the ER cholesterol homeostasis was not influenced by liver Ces1d deficiency, and the unaltered hepatic cholesterol level in LKO mice was not due to compensation of cholesterol synthesis regulated by SREBP2.

It has been reported recently that a group of Aster proteins, which are encoded by LXR target gene *Gramd1*, are responsible for the transport of HDL-derived cholesterol from the plasma membrane to ER (44), where bile acid synthesis, cholesterol esterification, and regulation of cholesterol synthesis occur. The expression of *Gramd1a* and *Gramd1c* encoding two liver Aster isoforms, Aster-a and Aster-c, was not different between Lox and LKO mice (Fig. 4).

### Inactivation of Ces1d in the liver did not lead to hepatic CE accumulation

**Fig. 5:**
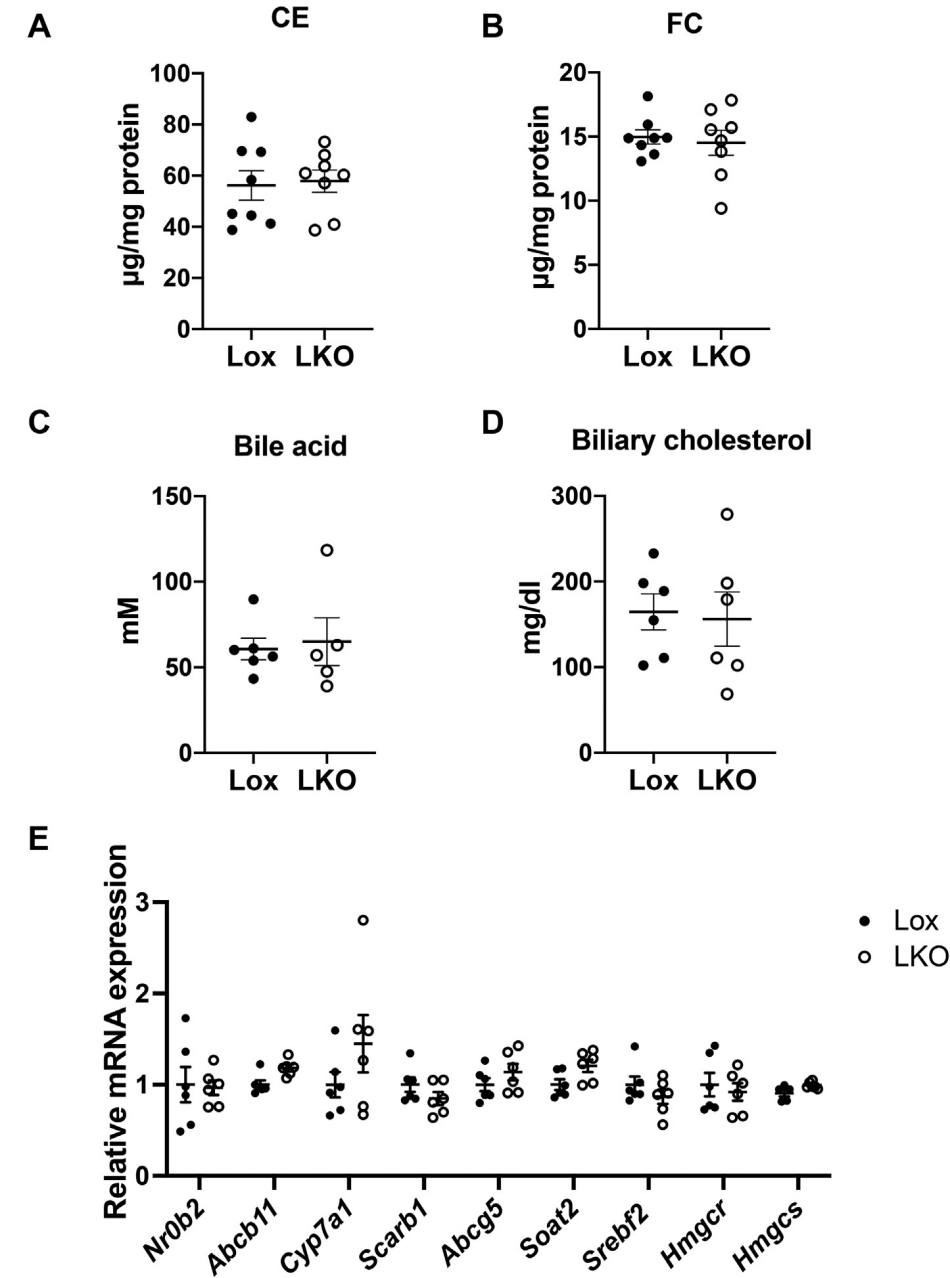
High-fat, high-cholesterol diet (Western-type diet, WTD) feeding does not cause CE accumulation in the liver of liver-specific Ces1d-KO mice. A: CE and (B) FC levels in the liver of Lox and LKO mice after 2 weeks of WTD feeding. C: Bile acid and (D) cholesterol levels in the gallbladder bile of Lox and LKO mice after 2 weeks of WTD feeding. E, mRNA expression of genes involved in bile acid metabolism and cholesterol metabolism in the liver of Lox and LKO mice after 2 weeks of WTD feeding. Ces1d, carboxylesterase 1d; FC, free cholesterol; LKO, liver-specific Ces1d KO; Lox, *Ces1d*^*flox/flox*^.

LKO mice on chow diet did not exhibit accumulation of CE in the liver (Fig. 2D). To further investigate whether Ces1d contributes to liver CE hydrolase activity, we challenged Ces1d-deficient mice with WTD, which contained high cholesterol content. We hypothesized that if Ces1d possesses substantial CE hydrolase activity, inactivation of hepatic Ces1d would lead to dramatic CE accumulation in the liver after a high-cholesterol diet challenge. After two weeks of WTD feeding, Lox mice showed about 60-fold increase in hepatic CE compared with the same genotype group in the chow-fed condition (Fig. 5A vs. Fig. 2D), and LKO mice had similar liver CE and FC concentrations compared with Lox mice after 2 weeks of WTD feeding (Fig. 5A, B), which suggested that Ces1d does not play a vital role in the hydrolysis of hepatic CE to FC.

Lox mice and LKO mice exhibited comparable bile acid and sterol concentrations in the bile (Fig. 5C, D), which suggested that in the condition of WTD feeding, the inactivation of liver Ces1d did not alter biliary excretion capacity of cholesterol.

Expression of genes encoding proteins that participate in bile acid synthesis and secretion (SHP, ABCB11, and CYP7A1) was not altered in the LKO mice fed with WTD (Fig. 5E). Similarly, expression of genes encoding SR-BI, ABCG5, ACAT2, and SREBP2, HMG-CoA reductase, and HMG-CoA synthase was not different between Lox and LKO mice (Fig. 5E). These data suggested that hepatic cholesterol and bile acid metabolism was not changed in the liver of LKO mice in the condition of WTD feeding.

### Influence of Ces1d deficiency on expression of other carboxylesterases in the liver

To address whether ablation of Ces1d resulted in a change of expression of genes encoding other hepatic carboxylesterases, we measured the expression of *Ces1* and *Ces2* gene families in Lox and LKO mice. In chow diet–fed mice, no significant change in hepatic *Ces1 and Ces2* gene expression was observed in LKO mice (supplemental Fig. S2A). After 2 weeks of WTD feeding, several liver carboxylesterase genes, especially genes encoding carboxylesterase 1 isoforms (Ces1a, b, c, e, f), showed increased expression in LKO mice, suggesting compensation for Ces1d deficiency in diet challenge (supplemental Fig. S2B).

## Discussion

RCT is a pivotal pathway that removes excess cholesterol from peripheral tissues and transports it to the liver for excretion into bile and feces. This route is the major process by which HDL exerts protection against atherosclerosis (45). Lipid poor pre-β-HDL secreted from the liver and intestine initializes this process by taking up excess unesterified cholesterol from cells via ABCA1-mediated efflux, and mature HDL can further acquire unesterified cholesterol from cells via ABCG1-mediated efflux. Cholesterol acquired by HDL through efflux is esterified to CE by plasma LCAT. HDL-CE is then selectively taken up by the liver via SR-B1 (3) and hydrolyzed, and the resulting cholesterol can be exported to bile either in its unesterified form or as a bile acid, or esterified by ACAT to CE, which can be stored in LDs or secreted in VLDL. Hydrolysis of CE is the rate-limiting step of ABCA1/ABCG1-mediated cholesterol efflux from macrophages (14) and hydrolysis of CE in the liver is necessary for cholesterol export into bile. Although CE hydrolase activity is critical for both the first step and the last step of RCT, the enzyme(s) responsible for the catalytic activity is/are still not fully elucidated. Ces1d (murine)/CES1 (human) has been reported to exhibit CE hydrolase activity in the macrophage (human) and liver and has been postulated to be involved in the RCT process (9, 10, 11, 12). However, the role of Ces1d/CES1 as a CE hydrolase has been disputed (15, 16, 17).

In the present study, we utilized LKO mice and performed an in vivo RCT assay by injecting mice with HDL-[^3^H]CE. The results suggest that absence of hepatic Ces1d did not affect the rate of uptake of HDL-CE into the liver, the conversion of HDL-CE into FC, or the elimination of HDL-derived cholesterol from the liver into bile and subsequently feces. Thus, Ces1d does not appear to play an important role in HDL-to-feces RCT flux in mice. Furthermore, LKO mice did not exhibit further CE accumulation in the liver compared with Lox mice when fed with either chow diet or WTD, which suggested that liver CE hydrolase activity was not significantly impaired by Ces1d inactivation. This conclusion was supported by the in vitro assay showing that Ces1d did not exhibit significant CE hydrolase activity, which is also in agreement with earlier studies (16, 17). In addition, ablation of Ces1d expression did not result in significant changes in expression of genes regulating cholesterol synthesis, uptake, and efflux. After 2 weeks of WTD feeding, several carboxylesterase genes encoding carboxylesterase 1 isoforms showed increased expression in the liver of LKO mice, suggesting compensation for Ces1d deficiency in diet challenge. However, owing to the lack of CE hydrolase activity of Ces1d observed in this and other studies (16, 17), the elevated expression of hepatic carboxylesterases is unlikely to compensate for the loss of CE hydrolase activity but may be related to other functions of Ces1d. The roles of other carboxylesterases beside Ces1d/CES1 in hepatic cholesterol metabolism have not yet been studied.

Bie *et al.* (13) reported that after 16 weeks of WTD feeding, Ces1d-deficient *Ldlr*^*−/−*^ mice exhibit decreased HDL-to-feces RCT and increased atherosclerosis without changes in liver lipids or plasma cholesterol levels. Although the distinct role of Ces1d in the in vivo RCT was observed, the abovementioned study (13) was performed with a different animal model and feeding regimen from this study. LDLr deficiency and long-term (16 weeks) WTD feeding would cause more severe hepatic lipid accumulation and hypercholesterolemia in mice. However, we previously reported (20) alleviated hyperlipidemia and atherosclerosis in Ces1d-deficient *Ldlr*^*−/−*^ mice after 12 week WTD feeding. And, if HDL-CE taken up into the liver and/or intracellular CE stores could not be converted to FC and/or bile acids for excretion because of Ces1d deficiency, excessive accumulation of cholesterol in vivo would be expected and this was not observed in the study (13), and therefore, the fate of cholesterol in that study is unclear.

In support of our findings, the lack of direct involvement of Ces1d/CES1 in the catabolism of HDL-derived or LD-stored CE in the liver is also supported by the localization of Ces1d/CES1 in the cell. Ces1d/CES1 are disulfide-bonded glycoproteins localized in the lumen of the ER (22, 23, 24, 25, 26). The proteins are targeted to this compartment by cleavable signal sequences and are retained in the ER by C-terminal ER-retrieval domains (HVEL in Ces1d and HIEL in CES1) (24). The location of HDL-derived CE hydrolysis after its uptake by the SR-BI is unclear but would not be expected to take place in the lumen of the ER because this would necessitate the transfer of CE from cytosol across the ER bilayer.

Other lipases have also been reported to participate in the cellular CE hydrolysis. Two other neutral CE hydrolases that have been reported in macrophages are HSL (46) and KIAA1363 [also called neutral cholesterol ester hydrolase 1 or arylacetamide deacetylase-like 1] (47). In a direct comparison study, ablation of HSL in mouse macrophages decreased cholesterol efflux, whereas inactivation of KIAA1363 did not affect CE hydrolase activity and cholesterol efflux in mouse macrophages (48), which challenged the function of KIAA1363 in macrophage CE metabolism. However, the expression of HSL was reported to be absent in human macrophages (49), which questions its role as a CE hydrolase in the human macrophage. In addition to neutral CE hydrolases, it has been reported that lipophagy and lysosomal acid lipase could mediate CE hydrolysis and produce FC for cholesterol efflux in CE-laden foam cells (50), thus providing an alternative mechanism for macrophage CE hydrolysis.

In the liver, additional CE hydrolase candidates need to be explored. HSL is involved in HDL-CE hydrolysis in mouse adrenal cortex (51, 52), but its role in liver HDL-derived CE metabolism has not been systemically elucidated. HSL would not be expected to play a major role in hepatic CE metabolism in humans because like in human macrophages, HSL is not expressed in the human liver. The potential role of lysosomal acid lipase in HDL-CE hydrolysis in the liver has not yet been reported. Arylacetamide deacetylase (AADAC) is another lipase that exhibits CE hydrolase activity and is expressed in the liver (53, 54). AADAC is a type II ER membrane glycoprotein with the active site oriented toward the ER lumen (55). In a proteomic study exploring LD-associated proteins in the mouse liver, some AADAC was found to coisolate with LDs (56), suggesting that AADAC could be localized at ER-LD contact sites (57). However, the detailed role of AADAC in RCT and CE hydrolysis is currently unknown.

CES1/Ces1d has been reported as a TAG hydrolase (58, 59, 60), and its roles in physiological processes that are related to TAG metabolism have been documented in several studies, including LD maturation (25) and VLDL assembly (7) in the liver, and also lipolysis in WAT (61).

In conclusion, our study suggests that Ces1d is not an important CE hydrolase catalyzing HDL-derived CE or stored CE turnover in the liver. Deficiency of Ces1d in the liver does not influence HDL-to-feces cholesterol excretion or liver CE storage. Future studies in this field should be directed to identification and characterization of other potential candidates of hepatic CE hydrolases.

## Data availability

All data are contained within the article.

## Supplemental data

This article contains supplemental data.

## Conflict of interest

The authors declare that they have no conflicts of interest with the contents of this article.

## References

[1] Libby P., Theroux P. (2005). Pathophysiology of coronary artery disease. Circulation.

[2] Rousset X., Vaisman B., Amar M., Sethi A.A., Remaley A.T. (2009). Lecithin: cholesterol acyltransferase--from biochemistry to role in cardiovascular disease. Curr. Opin. Endocrinol. Diabet. Obes..

[3] Shimada A., Tamai T., Oida K., Takahashi S., Suzuki J., Nakai T., Miyabo S. (1994). Increase in neutral cholesteryl ester hydrolase activity produced by extralysosomal hydrolysis of high-density lipoprotein cholesteryl esters in rat hepatoma cells (H-35). Biochim. Biophys. Acta.

[4] Lian J., Nelson R., Lehner R. (2018). Carboxylesterases in lipid metabolism: from mouse to human. Protein Cell.

[5] Quiroga A.D., Lehner R. (2018). Pharmacological intervention of liver triacylglycerol lipolysis: The good, the bad and the ugly. Biochem. Pharmacol..

[6] Lian J., Bahitham W., Panigrahi R., Nelson R., Li L., Watts R., Thiesen A., Lemieux M.J., Lehner R. (2018). Genetic variation in human carboxylesterase CES1 confers resistance to hepatic steatosis. Biochim. Biophys. Acta Mol. Cell Biol. Lipids.

[7] Wei E., Ben Ali Y., Lyon J., Wang H., Nelson R., Dolinsky V.W., Dyck J.R., Mitchell G., Korbutt G.S., Lehner R. (2010). Loss of TGH/Ces3 in mice decreases blood lipids, improves glucose tolerance, and increases energy expenditure. Cell Metab..

[8] Lian J., Wei E., Groenendyk J., Das S.K., Hermansson M., Li L., Watts R., Thiesen A., Oudit G.Y., Michalak M., Lehner R. (2016). Ces3/TGH deficiency attenuates steatohepatitis. Sci. Rep..

[9] Zhao B., Song J., Chow W.N., St Clair R.W., Rudel L.L., Ghosh S. (2007). Macrophage-specific transgenic expression of cholesteryl ester hydrolase significantly reduces atherosclerosis and lesion necrosis in Ldlr mice. J. Clin. Invest..

[10] Ghosh S., St Clair R.W., Rudel L.L. (2003). Mobilization of cytoplasmic CE droplets by overexpression of human macrophage cholesteryl ester hydrolase. J. Lipid Res..

[11] Zhao B., Song J., Ghosh S. (2008). Hepatic overexpression of cholesteryl ester hydrolase enhances cholesterol elimination and in vivo reverse cholesterol transport. J. Lipid Res..

[12] Bie J., Wang J., Yuan Q., Kakiyama G., Ghosh S.S., Ghosh S. (2014). Liver-specific transgenic expression of cholesteryl ester hydrolase reduces atherosclerosis in Ldlr-/- mice. J. Lipid Res..

[13] Bie J., Wang J., Marqueen K.E., Osborne R., Kakiyama G., Korzun W., Ghosh S.S., Ghosh S. (2013). Liver-specific cholesteryl ester hydrolase deficiency attenuates sterol elimination in the feces and increases atherosclerosis in ldlr-/- mice. Arterioscler. Thromb. Vasc. Biol..

[14] Ouimet M., Marcel Y.L. (2012). Regulation of lipid droplet cholesterol efflux from macrophage foam cells. Arterioscler. Thromb. Vasc. Biol..

[15] Igarashi M., Osuga J., Uozaki H., Sekiya M., Nagashima S., Takahashi M., Takase S., Takanashi M., Li Y., Ohta K., Kumagai M., Nishi M., Hosokawa M., Fledelius C., Jacobsen P. (2010). The critical role of neutral cholesterol ester hydrolase 1 in cholesterol removal from human macrophages. Circ. Res..

[16] Okazaki H., Igarashi M., Nishi M., Tajima M., Sekiya M., Okazaki S., Yahagi N., Ohashi K., Tsukamoto K., Amemiya-Kudo M., Matsuzaka T., Shimano H., Yamada N., Aoki J., Morikawa R. (2006). Identification of a novel member of the carboxylesterase family that hydrolyzes triacylglycerol: a potential role in adipocyte lipolysis. Diabetes.

[17] Sakai K., Igarashi M., Yamamuro D., Ohshiro T., Nagashima S., Takahashi M., Enkhtuvshin B., Sekiya M., Okazaki H., Osuga J., Ishibashi S. (2014). Critical role of neutral cholesteryl ester hydrolase 1 in cholesteryl ester hydrolysis in murine macrophages. J. Lipid Res..

[18] Ross M.K., Borazjani A., Mangum L.C., Wang R., Crow J.A. (2014). Effects of toxicologically relevant xenobiotics and the lipid-derived electrophile 4-hydroxynonenal on macrophage cholesterol efflux: silencing carboxylesterase 1 has paradoxical effects on cholesterol uptake and efflux. Chem. Res. Toxicol..

[19] Mangum L.C., Hou X., Borazjani A., Lee J.H., Ross M.K., Crow J.A. (2018). Silencing carboxylesterase 1 in human THP-1 macrophages perturbs genes regulated by PPARgamma/RXR and RAR/RXR: down-regulation of CYP27A1-LXRalpha signaling. Biochem. J..

[20] Lian J., Quiroga A.D., Li L., Lehner R. (2012). Ces3/TGH deficiency improves dyslipidemia and reduces atherosclerosis in Ldlr(-/-) mice. Circ. Res..

[21] Lian J., Wei E., Wang S.P., Quiroga A.D., Li L., Di Pardo A., van der Veen J., Sipione S., Mitchell G.A., Lehner R. (2012). Liver specific inactivation of carboxylesterase 3/triacylglycerol hydrolase decreases blood lipids without causing severe steatosis in mice. Hepatology.

[22] Robbi M., Beaufay H. (1988). Immunochemical characterization and biosynthesis of pI-6.4 esterase, a carboxylesterase of rat liver microsomal extracts. Biochem. J..

[23] Alam M., Vance D.E., Lehner R. (2002). Structure-function analysis of human triacylglycerol hydrolase by site-directed mutagenesis: identification of the catalytic triad and a glycosylation site. Biochemistry.

[24] Gilham D., Alam M., Gao W., Vance D.E., Lehner R. (2005). Triacylglycerol hydrolase is localized to the endoplasmic reticulum by an unusual retrieval sequence where it participates in VLDL assembly without utilizing VLDL lipids as substrates. Mol. Biol. Cell.

[25] Wang H., Wei E., Quiroga A.D., Sun X., Touret N., Lehner R. (2010). Altered lipid droplet dynamics in hepatocytes lacking triacylglycerol hydrolase expression. Mol. Biol. Cell.

[26] Wang H., Gilham D., Lehner R. (2007). Proteomic and lipid characterization of apolipoprotein B-free luminal lipid droplets from mouse liver microsomes: implications for very low density lipoprotein assembly. J. Biol. Chem..

[27] Hajjar D.P., Minick C.R., Fowler S. (1983). Arterial neutral cholesteryl esterase. A hormone-sensitive enzyme distinct from lysosomal cholesteryl esterase. J. Biol. Chem..

[28] Holm C., Osterlund T. (1999). Hormone-sensitive lipase and neutral cholesteryl ester lipase. Methods Mol. Biol..

[29] Gilham D., Lehner R. (2005). Techniques to measure lipase and esterase activity in vitro. Methods.

[30] Folch J., Lees M., Sloane Stanley G.H. (1957). A simple method for the isolation and purification of total lipides from animal tissues. J. Biol. Chem..

[31] Abreu S., Solgadi A., Chaminade P. (2017). Optimization of normal phase chromatographic conditions for lipid analysis and comparison of associated detection techniques. J. Chromatogr. A..

[32] Assmann G., Schriewer H., Schmitz G., Hagele E.O. (1983). Quantification of high-density-lipoprotein cholesterol by precipitation with phosphotungstic acid/MgCl2. Clin. Chem..

[33] Annema W., Nijstad N., Tolle M., de Boer J.F., Buijs R.V., Heeringa P., van der Giet M., Tietge U.J. (2010). Myeloperoxidase and serum amyloid A contribute to impaired in vivo reverse cholesterol transport during the acute phase response but not group IIA secretory phospholipase A(2). J. Lipid Res..

[34] Miettinen T.A., Ahrens E.H., Grundy S.M. (1965). Quantitative isolation and gas--liquid chromatographic analysis of total dietary and fecal neutral steroids. J. Lipid Res..

[35] Wang N., Arai T., Ji Y., Rinninger F., Tall A.R. (1998). Liver-specific overexpression of scavenger receptor BI decreases levels of very low density lipoprotein ApoB, low density lipoprotein ApoB, and high density lipoprotein in transgenic mice. J. Biol. Chem..

[36] Kozarsky K.F., Donahee M.H., Rigotti A., Iqbal S.N., Edelman E.R., Krieger M. (1997). Overexpression of the HDL receptor SR-BI alters plasma HDL and bile cholesterol levels. Nature.

[37] Graf G.A., Yu L., Li W.P., Gerard R., Tuma P.L., Cohen J.C., Hobbs H.H. (2003). ABCG5 and ABCG8 are obligate heterodimers for protein trafficking and biliary cholesterol excretion. J. Biol. Chem..

[38] Yu L., Hammer R.E., Li-Hawkins J., Von Bergmann K., Lutjohann D., Cohen J.C., Hobbs H.H. (2002). Disruption of Abcg5 and Abcg8 in mice reveals their crucial role in biliary cholesterol secretion. Proc. Natl. Acad. Sci. U. S. A..

[39] Yu L., Li-Hawkins J., Hammer R.E., Berge K.E., Horton J.D., Cohen J.C., Hobbs H.H. (2002). Overexpression of ABCG5 and ABCG8 promotes biliary cholesterol secretion and reduces fractional absorption of dietary cholesterol. J. Clin. Invest..

[40] Trauner M., Boyer J.L. (2003). Bile salt transporters: molecular characterization, function, and regulation. Physiol. Rev..

[41] Lu T.T., Makishima M., Repa J.J., Schoonjans K., Kerr T.A., Auwerx J., Mangelsdorf D.J. (2000). Molecular basis for feedback regulation of bile acid synthesis by nuclear receptors. Mol. Cell.

[42] Lehmann J.M., Kliewer S.A., Moore L.B., Smith-Oliver T.A., Oliver B.B., Su J.L., Sundseth S.S., Winegar D.A., Blanchard D.E., Spencer T.A., Willson T.M. (1997). Activation of the nuclear receptor LXR by oxysterols defines a new hormone response pathway. J. Biol. Chem..

[43] Brown M.S., Radhakrishnan A., Goldstein J.L. (2018). Retrospective on cholesterol homeostasis: the central role of Scap. Annu. Rev. Biochem..

[44] Sandhu J., Li S., Fairall L., Pfisterer S.G., Gurnett J.E., Xiao X., Weston T.A., Vashi D., Ferrari A., Orozco J.L., Hartman C.L., Strugatsky D., Lee S.D., He C., Hong C. (2018). Aster Proteins facilitate nonvesicular plasma membrane to ER cholesterol transport in mammalian cells. Cell.

[45] Rader D.J., Alexander E.T., Weibel G.L., Billheimer J., Rothblat G.H. (2009). The role of reverse cholesterol transport in animals and humans and relationship to atherosclerosis. J. Lipid Res..

[46] Small C.A., Goodacre J.A., Yeaman S.J. (1989). Hormone-sensitive lipase is responsible for the neutral cholesterol ester hydrolase activity in macrophages. FEBS Lett..

[47] Sekiya M., Osuga J., Nagashima S., Ohshiro T., Igarashi M., Okazaki H., Takahashi M., Tazoe F., Wada T., Ohta K., Takanashi M., Kumagai M., Nishi M., Takase S., Yahagi N. (2009). Ablation of neutral cholesterol ester hydrolase 1 accelerates atherosclerosis. Cell Metab..

[48] Buchebner M., Pfeifer T., Rathke N., Chandak P.G., Lass A., Schreiber R., Kratzer A., Zimmermann R., Sattler W., Koefeler H., Frohlich E., Kostner G.M., Birner-Gruenberger R., Chiang K.P., Haemmerle G. (2010). Cholesteryl ester hydrolase activity is abolished in HSL-/- macrophages but unchanged in macrophages lacking KIAA1363. J. Lipid Res..

[49] Contreras J.A., Lasuncion M.A. (1994). Essential differences in cholesteryl ester metabolism between human monocyte-derived and J774 macrophages. Evidence against the presence of hormone-sensitive lipase in human macrophages. Arterioscler. Thromb..

[50] Ouimet M., Franklin V., Mak E., Liao X., Tabas I., Marcel Y.L. (2011). Autophagy regulates cholesterol efflux from macrophage foam cells via lysosomal acid lipase. Cell Metab..

[51] Li H., Brochu M., Wang S.P., Rochdi L., Cote M., Mitchell G., Gallo-Payet N. (2002). Hormone-sensitive lipase deficiency in mice causes lipid storage in the adrenal cortex and impaired corticosterone response to corticotropin stimulation. Endocrinology.

[52] Kraemer F.B., Shen W.J., Harada K., Patel S., Osuga J., Ishibashi S., Azhar S. (2004). Hormone-sensitive lipase is required for high-density lipoprotein cholesteryl ester-supported adrenal steroidogenesis. Mol. Endocrinol..

[53] Trickett J.I., Patel D.D., Knight B.L., Saggerson E.D., Gibbons G.F., Pease R.J. (2001). Characterization of the rodent genes for arylacetamide deacetylase, a putative microsomal lipase, and evidence for transcriptional regulation. J. Biol. Chem..

[54] Tiwari R., Koffel R., Schneiter R. (2007). An acetylation/deacetylation cycle controls the export of sterols and steroids from S. cerevisiae. EMBO J..

[55] Lo V., Erickson B., Thomason-Hughes M., Ko K.W., Dolinsky V.W., Nelson R., Lehner R. (2010). Arylacetamide deacetylase attenuates fatty-acid-induced triacylglycerol accumulation in rat hepatoma cells. J. Lipid Res..

[56] Kramer D.A., Quiroga A.D., Lian J., Fahlman R.P., Lehner R. (2018). Fasting and refeeding induces changes in the mouse hepatic lipid droplet proteome. J. Proteomics.

[57] Mishra S., Khaddaj R., Cottier S., Stradalova V., Jacob C., Schneiter R. (2016). Mature lipid droplets are accessible to ER luminal proteins. J. Cell Sci..

[58] Alam M., Ho S., Vance D.E., Lehner R. (2002). Heterologous expression, purification, and characterization of human triacylglycerol hydrolase. Protein Expr. Purif..

[59] Lehner R., Vance D.E. (1999). Cloning and expression of a cDNA encoding a hepatic microsomal lipase that mobilizes stored triacylglycerol. Biochem. J..

[60] Dolinsky V.W., Sipione S., Lehner R., Vance D.E. (2001). The cloning and expression of a murine triacylglycerol hydrolase cDNA and the structure of its corresponding gene. Biochim. Biophys. Acta.

[61] Soni K.G., Lehner R., Metalnikov P., O'Donnell P., Semache M., Gao W., Ashman K., Pshezhetsky A.V., Mitchell G.A. (2004). Carboxylesterase 3 (EC 3.1.1.1) is a major adipocyte lipase. J. Biol. Chem..

