# Supplementary material for: Carboxylesterase 1d (Ces1d) does not contribute to cholesteryl ester hydrolysis in the liver
Source: J Lipid Res. 2021 Jun 18;62:100093. doi: 10.1016/j.jlr.2021.100093 (PMC8287225; doi:10.1016/j.jlr.2021.100093)
Supplement: Supplemental Figs. S1 and S2 [file mmc1.pdf]

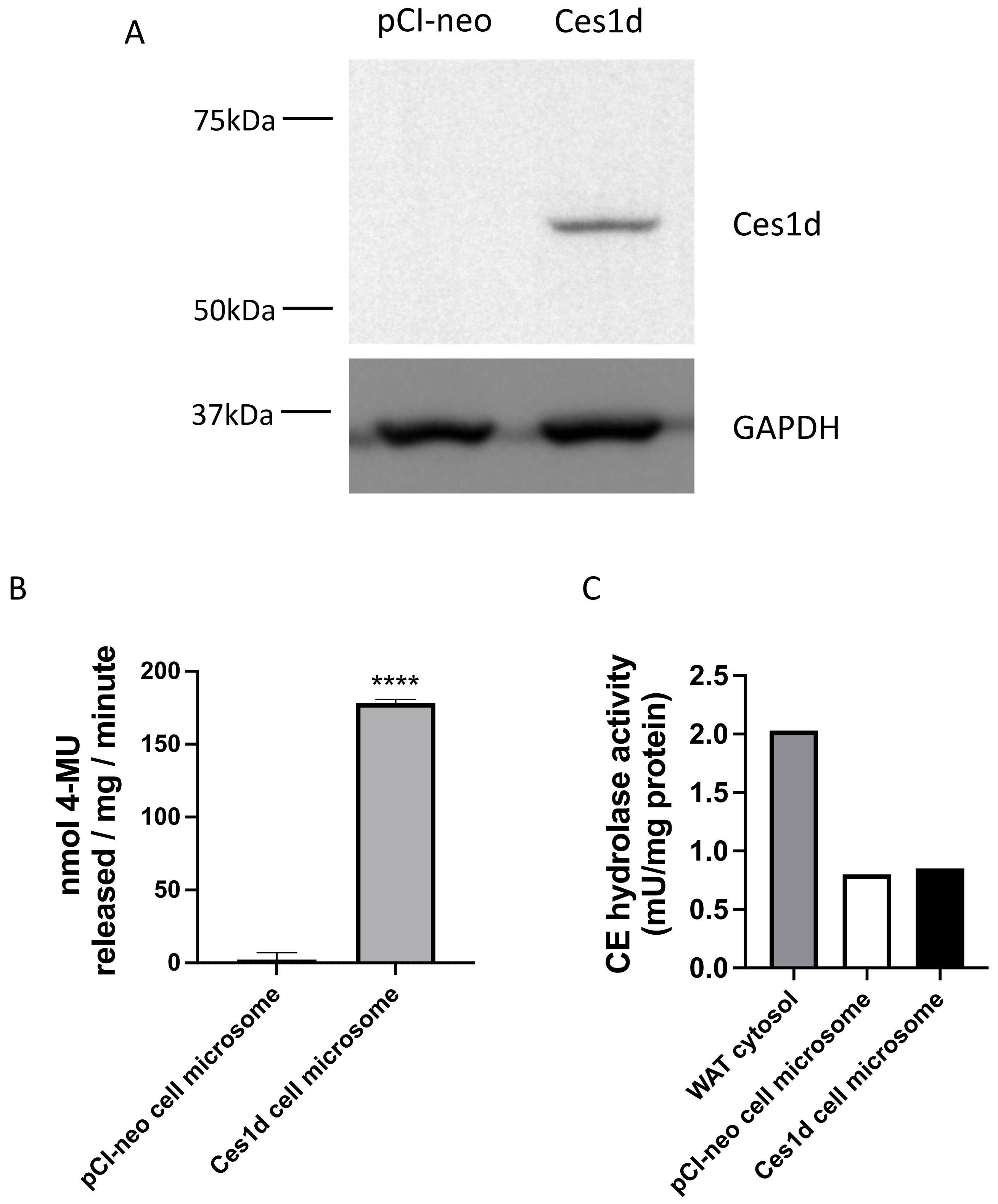

Figure S1. CE hydrolase activity was measured in Ces1d expressing cells. (A) Western blot to verify Ces1d expression with cell lysates. PCI-neo cell lysate was used as a negative control. (B) Lipase activity assay with microsomal fractions isolated from pCI-neo and Ces1d cells to verify expression of Ces1d in McA cells. (C) CE hydrolase activity in WAT cytosol and microsomal fractions isolated from pCI-neo and Ces1d expressing McA cells. ****P<0.0001.

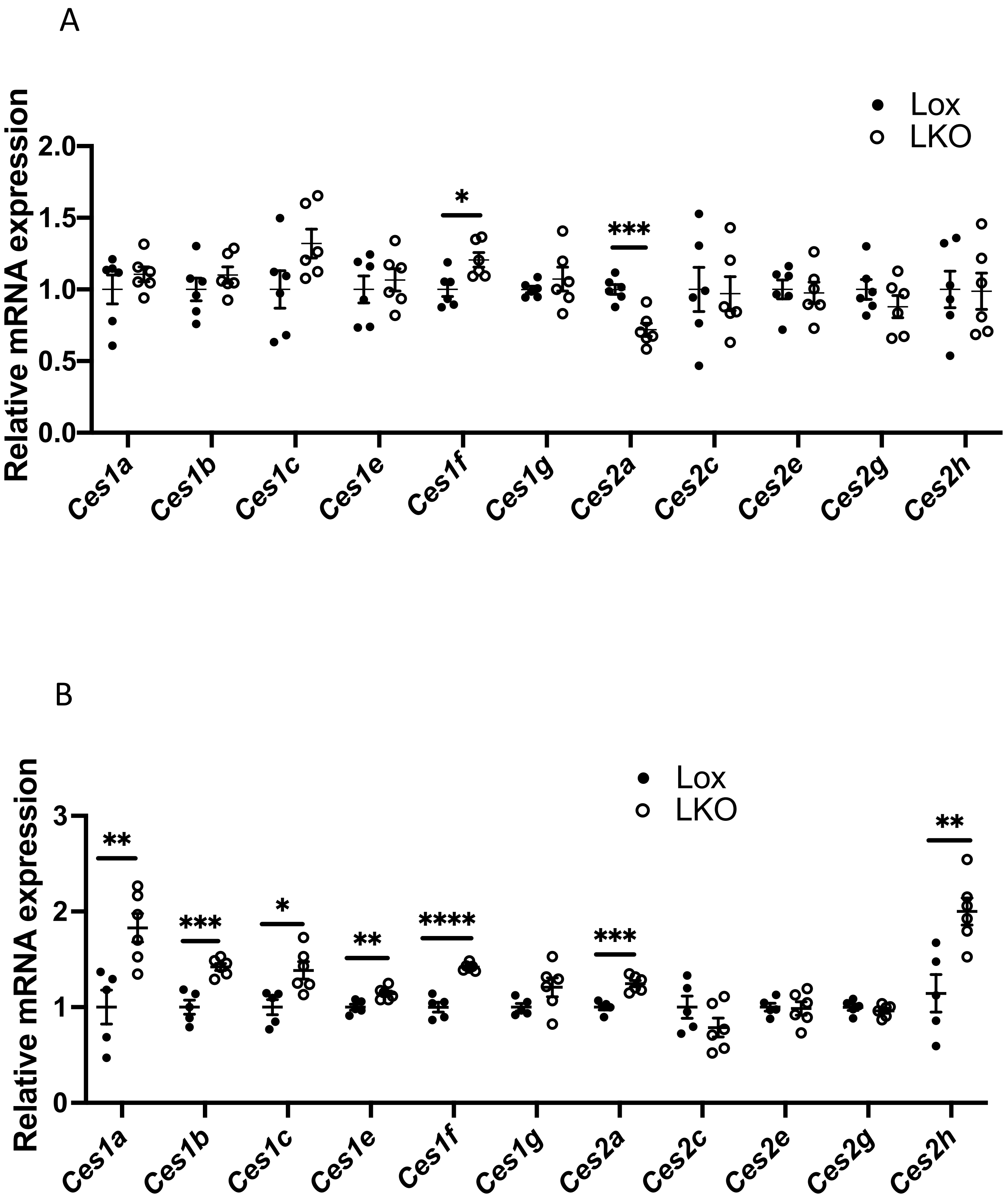

Figure S2. MRNA expression of liver carboxylesterases in Lox and LKO mice fed with (A) chow diet, and (B) WTD for two weeks. *P<0.05, **P<0.01, ***P<0.001, ****P<0.0001.
