# Supplementary material for: Carboxylesterase 1d (Ces1d) does not contribute to cholesteryl ester hydrolysis in the liver
Source: J Lipid Res. 2021 Jun 18;62:100093. doi: 10.1016/j.jlr.2021.100093 (PMC8287225; doi:10.1016/j.jlr.2021.100093)
Supplement: Supplemental Tables S1 and S2 [file mmc2.docx]

Supplementary Table 1. Primers used for quantitative PCR analysis

| **Gene** | **Sequence** |
| --- | --- |
| *Abcg5* | F: 5’- CTG CTC GCC TAC GTG CTA -3’ |
|  | R: 5’- ATC TGG CAA CTT CAG GAT ACA A -3’ |
| *Abcg11* | F: 5’- CAG TGG GTG TGG TAA AAG CA -3’ |
|  | R: 5’- TGC TGT CGT GAC CAT CTA TCA -3’ |
| *Cyp7a1* | F: 5’- ACA CCA TTC CTG CAA CCT TC -3’ |
|  | R: 5’- TCT TGG CCA GCA CTC TGT AA - 3’ |
| *Cyp8b1* | F: 5’- GCA GCA CTG AAT ACC CAT CC -3’ |
|  | R: 5’- TCT GAG AGC TGG GGA GAG G -3’ |
| *Cyp27a1* | F: 5’- CTT TCC TGA GCT GCT TTT GG -3’ |
|  | R: 5’- CAC CAG TCA CTT CCT TGT GC -3’ |
| *Hmgcr* | F: 5’- TGG GCA TGA ACA TGA TCT CTA -3’ |
|  | R: 5’- GGC TTC ACA AAC CAC AGT C -3’ |
| *Gramd1a* | F: 5’- TCT CGG ACT CTC CGT TCC T -3’ |
|  | R: 5’- CAA GGG CTC AAG GTC ACA TC -3’ |
| *Gramd1c* | F: 5’- ACC GTG GAC AGT TAT GAC ACC -3’ |
|  | R: 5’- ACA TTC AGC AGA ACG AGC AG -3’ |
| *Nr0b2* | F: 5’- CGA TCC TCT TCA ACC CAG AT -3’ |
|  | R: 5’- AGC CTC CTG TTG CAG GTG T -3’ |
| *Nr1h3* | F: 5’- CAG AAG AAC AGA TCC GCT TGA AG -3’ |
|  | R: 5’- TGC AAT GGG CCA AGG CGT GAC -3’ |
| *Scarb1* | F: 5’- TGG CAT TCA GAG CAG TGT AAC -3’ |
|  | R: 5’- CCG TTG GCA AAC AGA GTA TC -3’ |
| *Soat2* | F: 5’- GAG AAG GGA ACG CAA GGA C -3’ |
|  | R: 5’- TGC TTG CTC CAG AAA CTG TG -3’ |

| **Gene** | **Sequence** |
| --- | --- |
| *Ces1a* | F: 5’- CCC TAC AGA CCT GAC AAG CAA AGA -3’ |
|  | R: 5’- ATG CTC CAC CAG AGA GTA AAC CG -3’ |
| *Ces1b* | F: 5’- AGA TTC AGT GAC CGT CTT TGG -3’ |
|  | R: 5’- TGG CCA GAG GAG ATA AGA CAA -3’ |
| *Ces1c* | F: 5’- TTC GGG GCT CCA CTA TTA AA -3’ |
|  | R: 5’- GAG CAA AGT TGG CCC AGA - 3’ |
| *Ces1e* | F: 5’- AAA GAG TGA CAG ATT GCC AGT G -3’ |
|  | R: 5’- TTG ATG CCC CAC CTA ACA CTA -3’ |
| *Ces1f* | F: 5’- GCC TTA CAT TGT GGG AAT CAA -3’ |
|  | R: 5’- AGC TGG TAG AAA TCC CGT CA -3’ |
| *Ces1g* | F: 5’- TCG TGT CCC GTA GTC ACA GA -3’ |
|  | R: 5’- CAA AAC TTG GGC GAT ACT GAT -3’ |
| *Ces2a* | F: 5’- GCC ATT ATG CAG AGT GGA GTG -3’ |
|  | R: 5’- TCA CAA CCA GAT AGG TTG GCT A -3’ |
| *Ces2c* | F: 5’- GGC TGA ATG CTG GGT TCT T -3’ |
|  | R: 5’- TGG CCT CTG GTG AGT CCT -3’ |
| *Ces2e* | F: 5’- TGT GCT CCC TGA TCT TAT CTC C -3’ |
|  | R: 5’- GCT GCA CAG CCA GAT AGA TTG -3’ |
| *Ces2g* | F: 5’- TCT GAG GTG GTT TAC CAA ACG -3’ |
|  | R: 5’- CTC TTC ACT CTT GCC TCT CAG AC -3’ |
| *Ces2h* | F: 5’- GGG CTC CTG CTT CTC CTC -3’ |
|  | R: 5’- CCT GTG TGT GTG GTC CTG AT -3’ |

Supplementary Table 2. Primers used for carboxylesterases quantitative PCR analysis
